# Retracted: Side-by-Side Comparison of the Biological Characteristics of Human Umbilical Cord and Adipose Tissue-Derived Mesenchymal Stem Cells

**DOI:** 10.1155/2020/3176431

**Published:** 2020-09-23

**Authors:** BioMed Research International

BioMed Research International has retracted the article titled “Side-by-Side Comparison of the Biological Characteristics of Human Umbilical Cord and Adipose Tissue-Derived Mesenchymal Stem Cells” [1]. The article is one of four other articles [2–5] published by the same group of authors in 2012 and 2013. It was found to contain duplicated figures and data, where some of the cytokine expression data presented in Table 1 of [2] are repeated in Table 3 of [1], Table 1 of [4], and Table 1 of [5]. Details of the figure duplication are as follows:

- Figure 2 (a1 - a4) in [1] are the same as Figure 1d in [2] and Figure 2E in [3].
- Figure 4 (a1, b1) in [1] are the same as Figures 2F and 2G in [3] and Figure 3 and 4 in [4].
- Figure 6a in [1] is the same as Figure 2H in [3], but rotated, and Figure 2a in [2].
- Figure 2 (b1, b3) in [1] are the same as the 1st and 3rd panel of Figure 2 in [5].
- Figure 1a in [1] is the same as Figure 2B in [3].

The authors apologize for these errors and agree to the retraction of the article. The author's institution investigated our concerns and concluded that these errors were not the result of academic misconduct. The article is being retracted with the agreement of the journal and the editorial board due to concerns regarding the reliability of the data.
